# Host Response and Neo-Tissue Development during Resorption of a Fast Degrading Supramolecular Electrospun Arterial Scaffold

**DOI:** 10.3390/bioengineering5030061

**Published:** 2018-08-06

**Authors:** Renee Duijvelshoff, Nicole C. A. van Engeland, Karen M. R. Gabriels, Serge H. M. Söntjens, Anthal I. P. M. Smits, Patricia Y. W. Dankers, Carlijn V. C. Bouten

**Affiliations:** 1 Department of Biomedical Engineering, Eindhoven University of Technology, 5600 MB Eindhoven, The Netherlands; r.duijvelshoff@tue.nl (R.D.); N.C.A.v.Engeland@tue.nl (N.C.A.v.E.); karen.gabriels@outlook.com (K.M.R.G.); A.I.P.M.Smits@tue.nl (A.I.P.M.S.); P.Y.W.Dankers@tue.nl (P.Y.W.D.); 2 Institute for Complex Molecular Systems (ICMS), 5600 MB Eindhoven, The Netherlands; 3 SyMO-Chem B.V., 5600 MB Eindhoven, The Netherlands; S.Sontjens@tue.nl

**Keywords:** biomaterials, vascular graft, host response, macrophage, tissue engineering

## Abstract

In situ vascular tissue engineering aims to regenerate vessels “at the target site” using synthetic scaffolds that are capable of inducing endogenous regeneration. Critical to the success of this approach is a fine balance between functional neo-tissue formation and scaffold degradation. Circulating immune cells are important regulators of this process as they drive the host response to the scaffold and they play a central role in scaffold resorption. Despite the progress made with synthetic scaffolds, little is known about the host response and neo-tissue development during and after scaffold resorption. In this study, we designed a fast-degrading biodegradable supramolecular scaffold for arterial applications and evaluated this development in vivo. Bisurea-modified polycaprolactone (PCL2000-U4U) was electrospun in tubular scaffolds and shielded by non-degradable expanded polytetrafluoroethylene in order to restrict transmural and transanastomotic cell ingrowth. In addition, this shield prevented graft failure, permitting the study of neo-tissue and host response development after degradation. Scaffolds were implanted in 60 healthy male Lewis rats as an interposition graft into the abdominal aorta and explanted at different time points up to 56 days after implantation to monitor sequential cell infiltration, differentiation, and tissue formation in the scaffold. Endogenous tissue formation started with an acute immune response, followed by a dominant presence of pro-inflammatory macrophages during the first 28 days. Next, a shift towards tissue-producing cells was observed, with a striking increase in α-Smooth Muscle Actin-positive cells and extracellular matrix by day 56. At that time, the scaffold was resorbed and immune markers were low. These results suggest that neo-tissue formation was still in progress, while the host response became quiescent, favoring a regenerative tissue outcome. Future studies should confirm long-term tissue homeostasis, but require the strengthening of the supramolecular scaffold if a non-shielded model will be used.

## 1. Introduction

Recent developments in vascular tissue engineering strategies show a shift towards in situ vascular tissue engineering while using cell-free synthetic scaffolds that promote tissue regeneration inside the human body [1,2]. This in situ approach aims to harness the natural foreign body response towards wound healing process that enables the regeneration of vascular tissues. However, the physiological wound healing process involves a complex series of events that require strict regulation in order to prevent detrimental outcomes, such as fibrosis or chronic inflammation [3]. These events pose several scientific challenges for tissue engineers in designing scaffolds that are capable of supporting and guiding a favorable wound healing response via a pro-regenerative immune environment.

Despite the progress that has been made in the development of biodegradable polymeric scaffolds, the targeted regenerative process is not well understood. It is hypothesized that this regenerative process is inflammation-driven, and that early infiltration of immune cells is a determining factor in late tissue outcome [4,5]. Based on a previous study by Talacua et al. [6], we hypothesize that these infiltrating immune cells are endogenous circulating monocytes and macrophages, which initiate an inflammatory response by secreting cytokines and growth factors that subsequently attract progenitor cells that are capable of matrix formation. The source of these progenitor cells remains to be further elucidated. Potential sources of these progenitor cells include the circulation, the bone marrow, resident tissue cells, and macrophages that may transdifferentiate into tissue producing cells. In order to specify which changes in biomaterial design are needed to direct these cells towards tissue regeneration and to prevent adverse remodeling, it is of great importance to fully understand their interaction with the biomaterial during scaffold remodeling.

Several studies have investigated the regenerative potential of different synthetic polymeric scaffolds as tissue-engineered vascular grafts (TEVGs) in the arterial circulation of a small animal model [6,7,8,9,10,11,12]. Various biodegradable synthetic polymers were studied in this context, including poly(ε-caprolactone) (PCL), poly(glycerol sebacate) (PGS), poly(glycolic acid) (PGA), poly(lactic acid) (PLA), polyurethane (PU), and copolymers thereof. A disadvantage of these materials is that they lack the potential to easily adjust the material properties to match tissue formation by the host and to guide the regeneration process. 

A promising new class of highly tunable synthetic materials in vascular tissue engineering is supramolecular polymers, which are formed by mixing different building blocks that are non-covalently linked through directional and reversible secondary interactions. Due to the reversibility in the bonding, polymer properties, such as mechanics and degradation rate, can be modified to obtain a variety of different polymers with distinct material characteristics [13]. Moreover, it provides a powerful tool to customize these materials for specific vascular applications, e.g., by the incorporation of non-cell adhesive components, specific-cell attracting peptides, or bioactive moieties [14,15,16]. Due to the possibility to fine-tune their properties in such a controlled way, these supramolecular materials allow for better control of the regenerative process and the prevention of adverse remodeling. In an earlier study, we successfully used a slow-degrading electrospun supramolecular scaffold that is made of bisurea-modified polycarbonate to ensure mechanical functionality in in-situ tissue engineered heart valves and followed native-like tissue formation for up to 12 months [17]. In the current in vivo study, we designed a faster degrading electrospun supramolecular scaffold for arterial applications and evaluated it in vivo. Tubular scaffolds were electropun of bisurea-modified polycaprolactone (PCL2000-U4U), which is a biodegradable thermoplastic elastomer that exhibits strong and elastic properties [18]. As the polycaprolactone backbone degrades through hydrolysis, it is more susceptible to degradation than the polycarbonate backbone, allowing for faster degradation. The PCL2000-U4U scaffolds were shielded with low-porosity expanded polytetrafluoroethylene (ePTFE), which slows down the rapid transmural and transanastomotic ingrowth of cells from the adjacent tissue, a phenomenon common in small animals, but not in humans [19,20]. Most importantly, the shielding prevented graft failure permitting to study neo-tissue and host response development during scaffold resorption. Scaffolds were implanted as an interposition graft into the abdominal aorta in a rat model, and explanted at different time points up to 56 days after implantation to monitor sequential cell infiltration, differentiation (immunohistochemistry, gene expression), and tissue formation (histology).

## 2. Materials and Methods

### 2.1. Polymer Synthesis

**Figure 1:**
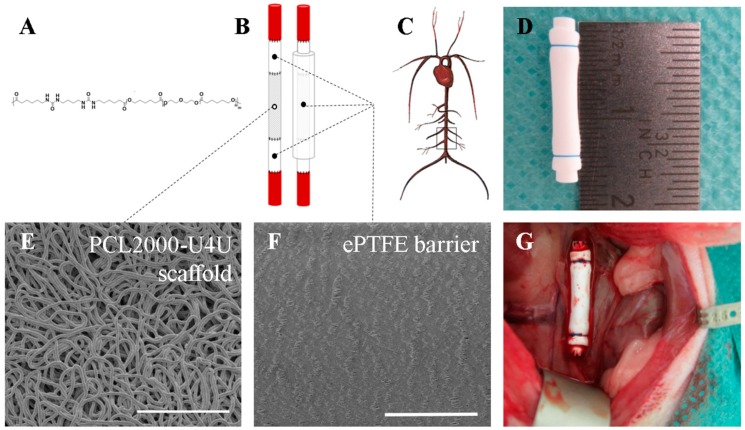
Scaffold composition and study design. (**A**) Chemical structure of PCL2000-U4U; (**B**) Schematic overview of tubular scaffold composition; (**C**) Schematic representation of the implantation site (infrarenal abdominal aorta of male Lewis rats); (**D**) Tubular scaffold prior to implantation; (**E**) Scanning Electron Microscopy (SEM) image of PCL2000-U4U scaffold. Scale bar represents 100 µm; (**F**) SEM image of ePTFE. Scale bar represents 100 µm; and, (**G**) Tubular scaffold in situ.

The polycaprolactone-bisurea PCL2000-U4U biomaterial (Figure 1A) was prepared, according to Wisse et al. [18]. The used batch had a weight average molecular weight of *M*_w_ = 74.4 kg/mol and a polydispersity of D = 2.3 (relative to polyethylene glycol standards), as assessed with gel permeation chromatography (GPC) while using a Varian/Polymer Laboratories PL-GPC 50 Plus instrument (Varian Inc., Palo Alto, CA, USA), equipped with a Shodex GPC KD-804 column (Shodex, Tokyo, Japan) that was operated at 50 °C, using DMF with 0.1% LiBr as the eluent.

### 2.2. Scaffold Fabrication

PCL2000-U4U (SyMO-Chem, Eindhoven, The Netherlands) was processed into a microfibrous porous isotropic scaffold by electrospinning. The polymer was dissolved in trichloromethane/methanol (99/1, w/w) (Sigma-Aldrich, St. Louis, MO, USA) at 12.5% (w/w) and stirred overnight. Following complete dissolution, the polymer solution was dispensed with the aid of a syringe pump (PHD 22/2000, Harvard Apparatus, Holliston, MA, USA) to a moving nozzle (0.8 mm internal diameter) placed at 15 cm distance from the rotating collecting mandrel (1.5 mm diameter) of a climate controlled electrospinning apparatus (EC-CLI, IME Technologies, Geldrop, The Netherlands). The electrospinning conditions were as follows: constant temperature of 23 °C, 30% relative humidity, 15 kV voltage on nozzle, 20 µL/min flow rate, 100 rpm collector rotation speed, and 15 min spin time. Electrospun scaffolds were kept under vacuum overnight to eliminate solvent remnants.

### 2.3. Scaffold Shielding and Sterilization

In order to shield the 8 mm long electrospun tubular PCL2000-U4U scaffold, an end-to-end anastomosis was made to a 4 mm long low-porosity ePTFE tube (Zeus Industrial Products Inc., Orangeburg, SC, USA) using interrupted 8-0 nylon sutures (Ethilon^®^; Ethicon, Johnson & Johnson, New Brunswick, NJ, USA) proximally and distally from the electrospun scaffold. In addition, a low-porosity ePTFE tube was mounted on the outer surface of the shielded electrospun tubular scaffold and tightened at both shielded sides using a single 6-0 suture ligature (Prolene^®^; Ethicon, Johnson & Johnson, New Brunswick, NJ, USA) (Figure 1B,D). The thickness of the ePTFE tube was 350 micrometer. All of the scaffolds were sterilized by Ethylene Oxide sterilization (Synergy Health, Venlo, The Netherlands) prior to implantation.

### 2.4. Assessment of PCL2000-U4U Scaffold and ePTFE Microstructure

Tubular PCL2000-U4U scaffolds and ePTFE (Zeus Industrial Products Inc, Orangeburg, SC, USA) were examined by scanning electron microscopy (SEM) (Quanta 600F, FEI, Hillsboro, OR, USA). Samples were visualized in high vacuum atmosphere with an electron beam of 1 kV. Inner tube diameter, wall thickness, and average fiber diameter or internodal distance (IND) were measured from SEM images using standard image processing software (ImageJ v1.48, U.S. NIH, Bethesda, MD, USA). To measure fiber diameter, at least 30 individual fibers of each sample were measured.

### 2.5. Experimental Animals

Sixty inbred male Lewis rats (Charles River Laboratories, Sulzfeld, Germany), weighing 306 ± 17 g, were used in this study. Animals were housed in pairs in individually ventilated cages at 20 °C and 50% humidity on a 12 h light-dark cycle with ad libitum access to standard chow and water. After one week of acclimatization, sixteen-millimeter long tubular scaffolds, composed of high-porosity PCL2000-U4U and shielded by low-porosity ePTFE, were implanted as an interposition graft into the animals’ infrarenal abdominal aorta. To study gene expression profiles and cellular infiltration and phenotype over time, scaffolds were explanted after 1 day (*n* = 9), 3 days (*n* = 9), 7 days (*n* = 8), 14 days (*n =* 8), 28 days (*n =* 13), and 56 days (*n =* 13). Part of the native abdominal aorta of each animal was explanted and used as control tissue. All of the animal experiments were reviewed and approved on 11 March 2014 by the Animal Ethics Committee of Maastricht University (The Netherlands) and conform to the guidelines for the use of laboratory animals, as formulated by the Dutch Law on animal experimentation. The project identification code of the animal study is: 2013-108.

### 2.6. Surgical Procedure

Prior to surgery, animals were given subcutaneous analgesia (buprenorphine 0.05 mg/kg). Operations were performed under general anesthesia (1.5–2.5% isoflurane) and under sterile conditions in spontaneously breathing animals while using an operation microscope (Leica Microsystems, Wetzlar, Germany). Body temperature was maintained at 37 °C using a heating pad. Animals were administered 25 IE of heparin subcutaneously prior to surgery. After a midline laparotomy, the aorta was separated from the inferior vena cava and surrounding tissues. The segment of the abdominal aorta between the renal arteries and the aortic bifurcation was mobilized (Figure 1C), collateral branches tied using 6-0 silk (Braun Aesculap, Tuttlingen, Germany), followed by aortic cross-clamping of the aorta between the renal arteries and the bifurcation with microvascular clamps. The aorta was transected and scaffolds were placed as an interposition graft with end-to-end anastomosis performed at both the proximal and the distal ends using interrupted 8-0 nylon sutures (Ethilon^®^; Ethicon, Johnson & Johnson, New Brunswick, NJ, USA). When the clamps were removed and hemostasis was achieved, the aorta was closely inspected to confirm pulsatile flow distal to the tubular scaffold. The abdomen was closed in two layers using 4-0 sutures (Vicryl^®^; Ethicon, Johnson & Johnson, New Brunswick, NJ, USA). Animals recovered in a recovery chamber at 30 °C and were assessed for evidence of acute failure, before returning to their cage. At the end of the day of surgery, animals were given subcutaneous analgesia (buprenorphine 0.05 mg/kg), which was continued twice-daily during the first three postoperative days. No anti-coagulation or anti-platelet therapy was given throughout the duration of the study. In addition to standard chow, animals received recovery dietgels (ClearH_2_O^®^, Westbrook, ME, USA) for three days postoperatively, in order to improve their recovery. 

On the predetermined day of sacrifice, animals were euthanized under isoflurane anesthesia by exsanguination. Animals were then systematically perfused with cold phosphate buffered saline (PBS) (Sigma-Aldrich, St. Louis, MO, USA), after which the scaffold and a native aorta specimen were carefully explanted. The external ePTFE tube was removed by cutting the two prolene sutures and by carefully cutting the ePTFE tube in a longitudinal direction.

### 2.7. Histology

Specimens were fixated in 3.7% formalin for 24 h at 4 °C, and embedded in optimal cutting temperature compound (OCT) (Tissue-Tek^®^, Sakura Finetek Europe B.V., Alphen aan den Rijn, The Netherlands). 5 µm thick sections were cut and mounted on Polysine^®^ glass slides (ThermoFischer Scientific, Waltham, MA, USA). Slides were washed in PBS and stained with Weigert’s Hematoxylin and Eosin (H&E) (Sigma-Aldrich, St. Louis, MO, USA), Elastica van Gieson (Merck, Darmstadt, Germany), and Masson’s trichome (Sigma-Aldrich, St. Louis, MO, USA). Stained slides were then dehydrated by quick exposure to a graded series of ethanol solutions (70–100%) and mounted in Entellan (Merck, Darmstadt, Germany). No xylene was used during the protocol to prevent damage to the polymeric fibers. Slides were visualised with a Zeiss Axio 681 Observer Z1 microscope (Carl Zeiss Microscopy GmbH, Jena, Germany).

### 2.8. Immunohistochemistry

Specimens were fixated in 3.7% formalin for 24 h at 4 °C, and then embedded in optimal cutting temperature compound (OCT) (Tissue-Tek^®^, Sakura Finetek Europe B.V., Alphen aan den Rijn, The Netherlands). 5 µm thick sections were cut and mounted on Polysine^®^ glass slides (ThermoFischer Scientific, Waltham, MA, USA). Slides were washed in PBS, and then placed in a 96 °C citrate antigen retrieval buffer (pH 6.1; DAKO) for 20 min. The buffer was then allowed to cool, after which slides were washed in a 0.05% PBS-Tween solution (PBS; Tween 20, Sigma-Aldrich, St. Louis, MO, USA). This was followed by a permeabilization step with 0.5% Triton-X100 (Merck, Darmstadt, Germany) in PBS for 15 min at room temperature. Slides were washed another time in 0.05% PBS-Tween solution. Blocking was performed by incubating slides in 5% normal goat serum (Invitrogen, Carlsbad, CA, USA) for 1 h at room temperature in a humidified chamber. Primary antibodies were prepared at the desired concentrations in 0.5% normal goat serum. Slides were incubated in primary antibody in a humidified chamber at 4 °C overnight. The primary antibodies used were: mouse anti-CD68 (1:400; AbDSerotec, Kidlington, UK), rabbit anti-inducible Nitric Oxide Synthase (iNOS) (1:400; Abcam, Cambridge, UK), mouse anti-CD163 (1:200; AbDSerotec, Kidlington, UK), mouse anti-α-Smooth Muscle Actin (α-SMA) (1:600; Sigma-Aldrich, St. Louis, MO, USA), and rabbit anti-von Willebrand Factor (vWF) (1:1200; Abcam, Cambridge, UK). The following day, slides were washed (0.05% PBS-Tween solution) and incubated with Alexa fluor 488/555 secondary antibodies (1:300; Invitrogen, Carlsbad, CA, USA) for 1 h at room temperature in a humidified chamber. Cell nuclei were stained with 4’,6-diamidino-2-phenylindole (DAPI). Incubation without primary antibody was included as negative controls. After washing (0.05% PBS-Tween solution), slides were mounted in Mowiol (Calbiochem, San Diego, CA, USA) and visualised with a Zeiss Axiovert fluorescence microscope (Carl Zeiss Microscopy GmbH, Jena, Germany).

### 2.9. Quantitative Immunohistochemical Analysis

Cellularity was studied using DAPI stainings. For each time point (day 7, day 14, day 28, and day 56), three explants were analyzed. Of each explant, four random areas were selected and digitally photographed using a Zeiss Axiovert fluorescence microscope (Carl Zeiss Microscopy GmbH, Jena, Germany). These images were converted into 8-bit gray-value images and further analyzed using Image J software (ImageJ v1.52, U.S. NIH, Bethesda, MD, USA). After selecting, duplicating, and measuring the area of interest, a threshold was set. Cell nuclei were separated by water shedding and subsequently counted using Image J software (ImageJ v1.52, U.S. NIH, Bethesda, MD, USA). Cell number was adjusted to measured area to allow for an equal comparison of the four images. The mean of the four images was then calculated. 

Quantification of CD68, iNOS, α-SMA, and vWF was studied using immunostainings of each marker. For each time point (day 7, day 14, day 28, and day 56), three explants were analyzed. Of each explant, four random areas were selected and digitally photographed using a Zeiss Axiovert fluorescence microscope (Carl Zeiss Microscopy GmbH, Jena, Germany). These images were converted into 8-bit gray-value images and further analyzed using Image J software (ImageJ v1.52, U.S. NIH, Bethesda, MD, USA). After selecting and duplicating the area of interest, a threshold was set. The area fraction of each marker was measured using Image J software (ImageJ v1.52, U.S. NIH, Bethesda, MD, USA). The mean of the four images was then calculated.

### 2.10. Gene Expression Analysis

Explanted specimens (scaffold or native aorta) were collected in Nalgene cryogenic vials (Sigma-Aldrich, St. Louis, MO, USA) containing RNA-free metal beads and stored at −80 °C. First, specimens were mechanically disrupted with a microdismembrator (Sartorius, Göttingen, Germany) three times for 30 s at 3000 rpm. Then, total RNA was extracted using TRIzol isolation, according to the manufacturer’s protocol (TRIzol, Life Technologies, Carlsbad, CA, USA). RNA concentration and purity were determined with a Qubit dsDNA HS Assay Kit (Thermo Scientific, Waltham, MA, USA) and a spectrophotometer (NanoDrop 1000™, Thermo Scientific, Waltham, MA, USA). cDNA was synthesized using SuperScript^®^ VILO™ cDNA Synthesis Kit (Thermo Scientific, Waltham, MA, USA). Quantitative real-time polymerase chain reaction (qPCR) was performed to evaluate the expression level of genes involved in inflammatory processes and tissue formation (Table S1) ((Sigma-Aldrich St. Louis, MO, USA); (Primerdesign, Southampton, UK)). Topoisomerase (DNA) I (TOP1), β-2 microglobulin (B2M), and ATP synthase, H^+^ transporting, mitochondrial F1 complex, beta polypeptide (ATP5B) were used as reference genes (Primerdesign, Southampton, UK). All of the samples were analyzed in duplicates. Data was analyzed using the delta-delta CT method. After normalization to the geometric mean of the three reference genes, the obtained values were further normalized to the native aorta control group for each time point. The groups at day 1 and 3 were not included in statistical analysis of gene expression, due to the limited amount of RNA extracted from the samples. The number of replicates included for analysis were: day 7 (*n =* 6), day 14 (*n =* 6), day 28 (*n =* 9), and day 56 (*n =* 6).

### 2.11. Statistical Analysis

Data are expressed as mean ± standard error of the mean. Due to a non-normal distribution of the data, data were analyzed with Kruskal-Wallis tests, followed by Dunn’s multiple comparison tests. Statistical differences were determined using Prism software (Graphpad Software v5.04, La Jolla, CA, USA) and considered to be significant for *p*-values < 0.05.

## 3. Results

### 3.1. Tubular Scaffolds are Composed of Microporous PCL2000-U4U and Dense ePTFE

SEM analysis confirmed that electrospinning resulted in isotropic fibrous tubular PCL2000-U4U scaffolds that consisted of non-fused, micrometer thick fibers (fiber diameter distribution of 3.5 ± 0.2 µm (mean ± standard deviation)) (Figure 1E). These results indicate a microporous structure, which is warranted to promote cellular infiltration for in situ vascular tissue engineering. The inner diameter of the scaffolds measured 1.3 mm, while the average wall thickness was 345 ± 37 µm (mean ± standard deviation). In contrast, the microstructure of the ePTFE used as shield was dense with an internodal distance of 10–20 µm (Figure 1F).

### 3.2. Histological Analysis Demonstrates Homogenous Cellular Infiltration and Tissue Formation

**Figure 2:**
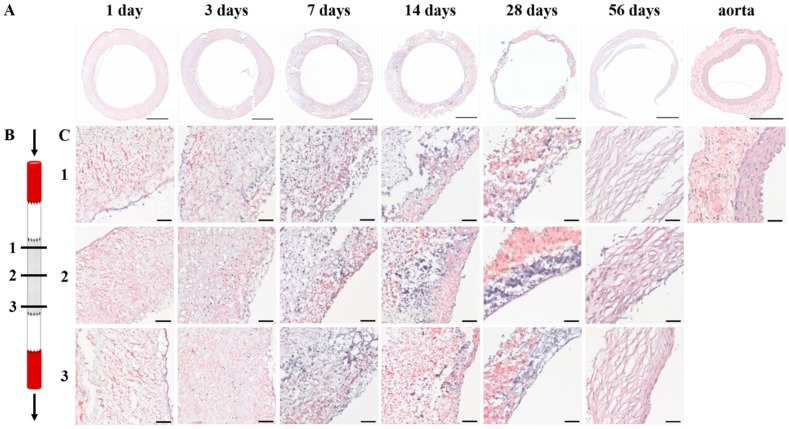
General morphology of explants. (**A**) Weigert’s H&E stainings of cross-sections of the explants after 1, 3, 7, 14, 28, and 56 days after implantation and native aorta. Scale bars represent 500 µm; (**B**) Schematic overview of the proximal (1), center (2), and distal (3) transection of the PCL2000-U4U scaffold. Arrows indicate blood flow; (**C**) Weigert’s H&E stainings of the explants after 1, 3, 7, 14, 28, and 56 days after implantation at the different locations in the scaffold, as illustrated in (**B**). Lumen is located at the right side of each picture. Scale bars represent 50 µm.

Histological evaluation of the explants showed that cells infiltrated the PCL2000-U4U scaffold from day 1 (Figure 2A,C). During the first days, cells populated the complete electrospun scaffold, leading to a homogenous cell distribution throughout the scaffold by day 7 (Figure 2A,C). General morphology of the explants changed with time, with a clear reduction in wall thickness 28 days after implantation (Figure 2A). At day 56, an initial tissue layering was observed, albeit still being very distinct from native control tissue, with no indication of scaffold present (Figure 2C). Throughout the duration of the study, no differences in tissue formation between proximal, center, and distal sites were observed (Figure 2C).

### 3.3. Pro-Inflammatory Macrophages Are the Dominant Cell Type during the First Month after Implantation

**Figure 3:**
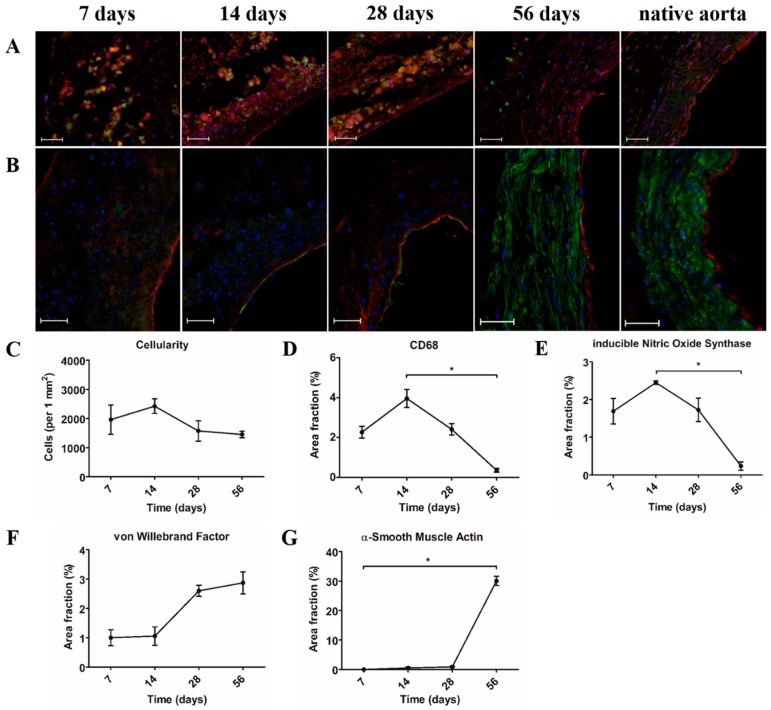
Cellular infiltration and phenotype. (**A**) Immunofluorescent co-staining of pan-macrophage marker CD68 (green) and pro-inflammatory macrophage marker anti-inducible Nitric Oxide Synthase (iNOS) (red). Lumen is located at the right side of each picture. Scale bars represent 50 µm; (**B**) Immunofluorescent co-staining of α-Smooth Muscle Actin (α-SMA) for smooth muscle cells (green) and von Willebrand Factor (vWF) for endothelium (red). Lumen is located at the right side of each picture. Scale bars represent 50 µm; (**C**) Cellularity in number of cells per square mm; (**D**) Area fraction in % of CD68; (**E**) Area fraction in % of iNOS; (**F**) Area fraction in % of vWF; and, (**G**) Area fraction in % of α-SMA. (**C**–**G**) * *p* < 0.05.

During the first month after implantation, mononuclear (CD68^+^) macrophages were the dominant cell type present in the PCL2000-U4U scaffold (Figure 3A). Macrophages were distributed throughout the scaffold, with a pronounced concentration at the sites of scaffold resorption. Macrophage subtypes were characterized by double-staining of CD68 (pan-macrophage marker) and iNOS (pro-inflammatory macrophage marker), or CD163 (anti-inflammatory macrophage marker), respectively. Most of the CD68^+^ cells co-expressed iNOS, indicating an abundant presence of pro-inflammatory macrophages. On the contrary, no CD163 expression was observed. After 56 days, when no scaffold could be observed, a major reduction in macrophages was seen (Figure 3A,D). Furthermore, the macrophages detected in the 56 days explants did not express iNOS (Figure 3A). An endothelial lining at the luminal side was already present after seven days (Figure 3B,F). While the first α-SMA^+^ cells were detected at day 14, only 56 days explants displayed a medial layer of circumferentially orientated α-SMA^+^ cells consistent with native tissue (Figure 3B,G).

### 3.4. Gene Expression Analysis Demonstrates Changes in Gene Expression Profile of Cells in the Scaffold over Time

**Figure 4:**
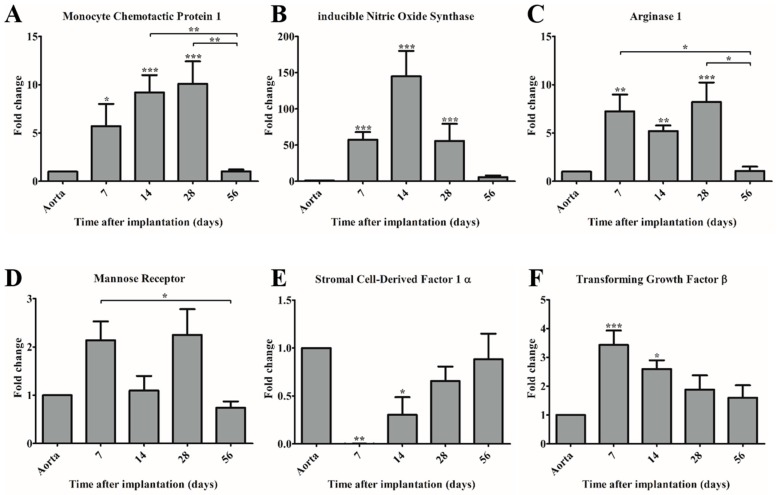
Gene expression of immunomodulating genes (**A**,**E**,**F**) and macrophage subset markers (**B**–**D**) in time compared to control (native aorta). Gene expression of (**A**) Monocyte Chemotactic Protein-1 (MCP-1); (**B**) anti-inducible Nitric Oxide Synthase (iNOS); (**C**) Arginase-1 (Arg-1); (**D**) Mannose Receptor (MR); (**E**) Stromal cell-Derived Factor 1α (SDF-1α); and, (**F**) Transforming Growth Factor-β (TGF-β). * *p* < 0.05 versus control, ** *p* < 0.01 versus control, *** *p* < 0.001 versus control.

Quantitative real-time PCR was performed to investigate the gene expression profile of recruited cells in the PCL2000-U4U scaffold over time. Monocyte Chemotactic Protein-1 (MCP-1) expression was significantly upregulated during the first month after implantation, when compared to the native control, with a peak value at day 28. In the consecutive month; however, a significant decrease in MCP-1 expression was observed, resulting in a gene expression level that is similar to native control (Figure 4A).

To assess the polarization state of the recruited macrophage population in the PCL2000-U4U scaffold, expression levels of genes that are attributed to either pro-inflammatory macrophages (iNOS), or anti-inflammatory macrophages (Arginase-1 (Arg-1), Mannose Receptor (MR)) were analyzed. Both iNOS and Arg-1 expression were significantly upregulated during the first month after implantation, with a peak value at day 14 (iNOS) and day 28 (Arg-1), respectively (Figure 4B,C). Of note, the peak value of iNOS expression was considerably higher than the peak value of both Arg-1 and MCP-1. After two months, the gene expression levels of iNOS and Arg-1 decreased and no significant difference between scaffold and native control could be detected, corresponding to the findings for MCP-1 expression (Figure 4A–C). In contrast to iNOS and Arg-1, MR expression remained relatively stable in time with no significant difference between scaffold and native control (Figure 4D). Gene expression levels of Stromal cell-Derived Factor 1α (SDF-1α) showed an increasing trend over time, but remained lower when compared to native control (Figure 4E).

Transforming Growth Factor-β (TGF-β) gene expression analysis revealed a significant increase at day 7 (peak value) and day 14 post-implantation, but no significant difference at later time points when compared to native control (Figure 4F). 

Expression of interleukin 1β, interleukin 4, interleukin 6, interleukin 13, and interferon-γ was undetectable (data not shown).

### 3.5. Matrix Genes Are Upregulated at Day 56 Post-Implantation Indicating Ongoing Neo-Tissue Formation

**Figure 5:**
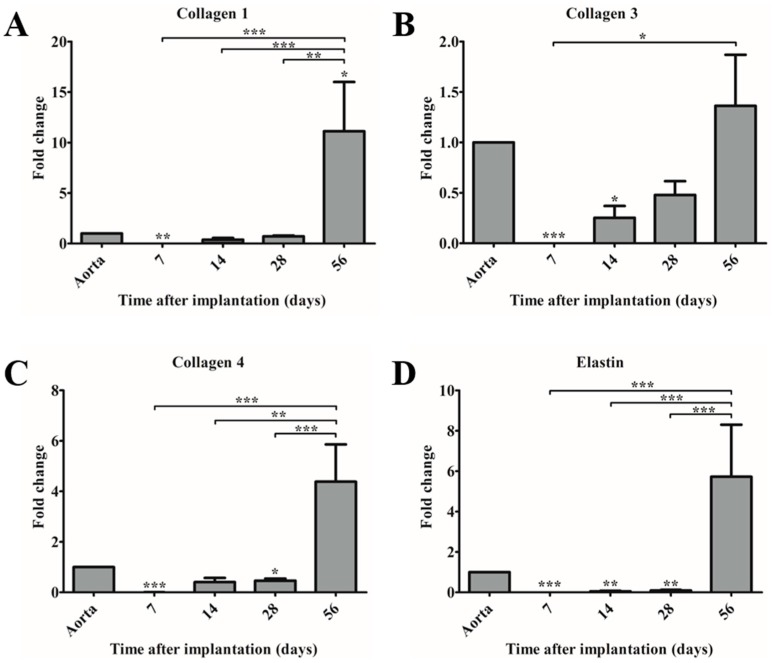
Gene expression of extracellular matrix components in time compared to control (native aorta). Gene expression of (**A**) collagen 1; (**B**) collagen 3; (**C**) collagen 4; and, (**D**) elastin. * *p* < 0.05 versus control, ** *p* < 0.01 versus control, *** *p* < 0.001 versus control.

Gene expression of collagen type I, III, IV, and elastin were analyzed at different time points in explants and compared to native aorta (Figure 5A–D). In time, an increase in gene expression of all genes was observed, of which collagen type I in the 56-day explants was significantly higher expressed than in native aorta (Figure 5A). However, all of the matrix genes remained upregulated in 56-day explants, suggesting ongoing neo-tissue formation and remodeling (Figure 5A–D).

### 3.6. 56-Day Explants Display Vascular Neo-Tissue Development with No Signs of Scaffold Present

**Figure 6:**
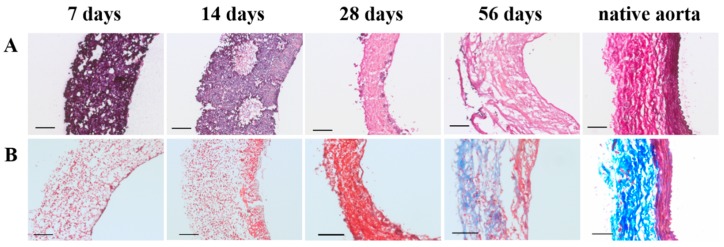
Scaffold degradation and extracellular matrix formation with time. (**A**) Elastica van Gieson staining (scaffold in purple, connective tissue in pink, and elastin fibers in dark purple). Lumen is located at the right side of each picture. Scale bars represent 100 µm; (**B**) Masson’s trichome staining (connective tissue in blue, cell nuclei in purple, and cytoplasm and muscle fibers in red). Lumen is located at the right side of each picture. Scale bars represent 100 µm.

Already after 7 days, scaffold resorption was observed, starting at small spots in the center of the PCL2000-U4U scaffold (Figure 6A). These spots expanded into larger areas (at day 14), where scaffold was replaced by extracellular matrix (ECM) (Figure 6A). After two months, no scaffold could be observed (Figure 6A). Although 56-day explants displayed the formation of vascular neo-tissue, no elastin fibers were detected (Figure 6A). 

As matrix production increased over time, an initial tissue layering was observed, which is characterized by cytoplasm and muscle fibers on the luminal side, surrounded by collagen on the non-luminal side (Figure 6B). Nonetheless, explants did not yet feature native-like morphology (Figure 6B).

## 4. Discussion

The aim of the current in vivo study was to investigate the regenerative process during the remodeling of a fast-degrading and tunable supramolecular PCL2000-U4U scaffold. The main objective was to improve the understanding of host-scaffold interaction during this process of regeneration in order to better define biomaterial design criteria for the PCL2000-U4U scaffolds. To specifically investigate neo-tissue formation and host response development during and after scaffold resorption, scaffolds were shielded by ePTFE, and implanted as interposition grafts into the abdominal aorta of Lewis rats.

**Figure 7:**
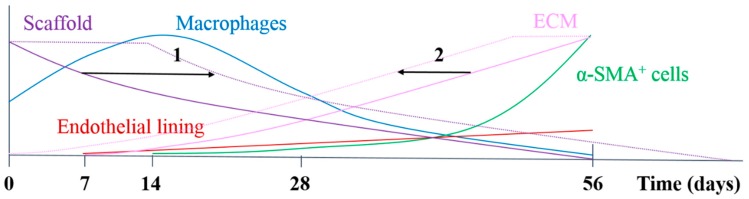
Schematic overview of the results of the study and options for chemical modification, either by decreasing degradation rate (1) or enhancement of tissue formation (2), e.g., by the addition of bioactive factors.

In the herein presented PCL2000-U4U scaffolds, a phased endogenous arterial regeneration was observed (Figure 7). The early phase after implantation was characterized by an acute inflammatory response, which is marked by the immediate influx of circulating immune cells and upregulation of immune genes. During the first 28 days after implantation, unexpected accelerated scaffold degradation was seen, which coincided with a dominant presence of pro-inflammatory macrophages in the scaffolds. Meanwhile, the start of ECM formation was observed. After 56 days, no scaffold could be observed and vascular neo-tissue had formed, and a shift from a dominant presence of pro-inflammatory macrophages towards tissue-producing cells was seen.

As in situ vascular tissue engineering aims to induce vascular regeneration directly at the target site, its successful application particularly depends on endogenous cells that are capable of neo-tissue formation. Moreover, circulating immune cells are considered the main regulators of this process, which makes them an important component in biomaterial design. Assessment of the explants by immunohistochemistry demonstrated that mononuclear macrophages (CD68^+^) were the dominant cell type that was present during the 28 days after implantation. Macrophages are highly plastic cells, which are capable of changing their polarization state in response to signals from their environment [21,22,23]. As such, they can exert both positive and negative effects on tissue remodeling, and a timely conversion from pro-inflammatory (M1) towards anti-inflammatory or wound healing (M2) phenotype has been proposed to be crucial in long-term tissue outcome [5,24]. In the present study, we observed that most of the CD68^+^ macrophages also stained positive for iNOS, indicating a pro-inflammatory phenotype, whereas CD163, which is an anti-inflammatory macrophage marker, could not be detected. The latter is consistent with previous findings in an animal model that restricted tissue ingrowth [6], although others have reported the presence of CD163 [8]. When no scaffold could be observed at day 56 post-implantation, a major reduction in macrophages was seen. Instead, a medial layer of circumferentially aligned SMA^+^ cells was present with vWF-expressing cells lining the lumen. 

The observed dominant presence of macrophages during the first 28 days was supported by gene expression analysis. Quantitative real-time PCR showed that MCP-1 expression levels significantly increased during the first 28 days, and decreased to control levels after 56 days. MCP-1 is considered to be a crucial immunomodulatory factor during tissue regeneration, as it enhances host monocyte recruitment in vivo [4,6]. The observed trend in MCP-1 expression correlated with the expression of macrophage subset markers iNOS and Arg1, indicating the recruitment of both pro- and anti-inflammatory macrophages into the PCL2000-U4U scaffolds during the first 28 days post-implantation.

Macrophages express iNOS at high levels after induction by cytokines or other inflammatory agents, in order to produce large amounts of nitric oxide as a part of the body’s defense system against pathogens or foreign materials [25]. This is of special interest in the current study, since it is known that PCL2000-U4U is prone to oxidative degradation, which involves the cell-generated production of reactive oxygen species, like nitric oxide [26]. In contrast to previous findings in a subcutaneous rat model, in which polycaprolactone discs were implanted, a much faster degradation of the electrospun PCL2000-U4U scaffold was observed when implanted as interposition graft into the rat abdominal aorta [18]. This is consistent with the findings of a recent study, which demonstrated that the biological response towards electrospun scaffolds is highly dependent on the implantation site [27]. We hypothesize that the rapid infiltration of (pro-inflammatory) macrophages, which was limited to the interface of the subcutaneously implanted solution cast films, significantly altered the degradation rate of the PCL2000-U4U scaffolds. The increased expression of iNOS during the first month may have even further contributed to an accelerated degradation rate, due to an increase in oxidative degradation. 

In addition to MCP-1, the expression of several other immunomodulating genes was examined. One of the genes of interest is SDF-1α, a chemokine that plays an important role in tissue repair signaling through the mobilization and homing of stem cells from the bone marrow to peripheral blood and the target tissue site [28]. On the gene level, an upregulation of SDF-1α was observed, although this was not significant. Interestingly, it seems that SDF-1α expression follows that of MCP-1 expression, which may imply that after the recruitment of macrophages, another progenitor cell type is attracted. Prior publications have shown that the incorporation of SDF-1α into scaffolds leads to an enhanced recruitment of autologous progenitor cells and subsequent improved tissue regeneration and angiogenesis [16,29]. Hence, SDF-1α represents a potential bioactive agent to be included into the PCL2000-U4U scaffolds to accelerate tissue regeneration.

Another gene analyzed was TGF-β, which plays an important role in vascular biology but it can also lead to chronic inflammation and fibrosis if it is not strictly regulated [30,31]. Prolonged elevation of TGF-β should therefore be avoided. In this study, it was observed that TGF-β expression was higher during the first 14 days after implantation when compared to native aorta. After 28 days, however, expression levels had declined to values that are equivalent to that of native aorta and remained constant throughout the course of the study, indicating a favorable down-regulation of TGF-β expression to avoid fibrosis. This is in line with recent studies, which demonstrated that timely scaffold degradation is essential to prevent fibrotic calcification of TEVGs in the arterial circulation [7,32].

Although the fast degradation of the PCL2000-U4U scaffold resulted in rapid neo-tissue formation, the matrix that was formed did not yet feature native-like morphology. For successful in vivo application, TEVGs should mimic native vessel morphology and mechanical properties. Whereas, collagen provides strength to the vascular wall, elastin enables the vascular wall to expand and contract passively with changes in pressure, thereby contributing to the compliance of the blood vessel [33]. Histological evaluation of the explants revealed the presence of collagen, but no signs of elastin fiber formation for up to 56 days. However, gene expression analysis did show a timely increase in the rate of elastin expression, indicating ongoing elastin formation. These results are in line with findings by Naito et al. [34], who described that the regeneration of a venous graft in mice consisted of an initial surge in fibrillar collagen production as a result of the foreign body response, while other ECM components (glycosaminoglycans, elastin, collagen IV) and functional maturation were observed in a later stage, after degradation of the scaffold [34,35]. One of the reasons we did not detect any elastin in the PCL2000-U4U scaffolds can be due to the time duration in which this study was conducted. However, we must also consider the negative effect of ePTFE shielding on the pulsatile loading of the scaffolds, hereby depriving cells from mechanical cues necessary for appropriate elastin production. In a recent study, Huang et al. [36] showed that cyclic biaxial stretching of tubular PGA scaffolds seeded with bovine smooth muscle cells enhanced the formation of mature elastin fibers in vitro, when compared to static controls. Moreover, lack of appropriate mechanotransduction was proposed to be the underlying cause of immature ECM formation in arterial TEVGs in mice [37].

The ePTFE shielding allowed for us to specifically investigate the neo-tissue formation and host response development during and after scaffold resorption. In addition, the shield prevented overall mechanical failure that could otherwise have led to aneurysmal dilatation or rupture, which has been previously reported [10]. When considering the accelerated degradation of the PCL2000-U4U scaffold, it must be acknowledged that the vascular neo-tissue could at any time have lacked the mechanical strength to withstand the arterial pressures it was exposed to if it had not been shielded by ePTFE.

Figure 7 provides a schematic overview of the results that were obtained in this study and illustrates potential ways to chemically modify the PCL2000-U4U scaffolds. Overall, the results of this study indicate that there was an imbalance between scaffold degradation kinetics with neo-tissue formation by the host, which could be the result of the restricted tissue in growth in the model used, leading to a delayed tissue formation. As transmural ingrowth from cells was hampered, this may have influenced this balance, as cells from transmural origin could not get in contact with the degradable tube to take part in the remodeling process. Ongoing debate emphasizes that the adventitial tissue might play an important role during vascular remodeling, and scaffolds should therefore be in contact with the surrounding tissue to investigate the complete remodeling process. Taking this into account, others have reported the successful transformation of a fast-degrading polymeric scaffold, composed of PGS and a PCL sheath, into compliant neo-arteries in which no PGS was present [8]. However, it remains to be investigated whether these results can be obtained in other larger animal models, due to interspecies differences in arterial regeneration [19]. In fact, even large variations within the same experimental animal group have been reported [38]. With a highly tunable material we intend to anticipate on the variability in arterial regeneration, by gaining a most favorable control over the regenerative process through optimal biomaterial design while using supramolecular chemistry.

## 5. Conclusions

In this in vivo study, we have specifically investigated the neo-tissue formation and host response development during the resorption of a supramolecular PCL2000-U4U scaffold for arterial regeneration in situ. During the course of the study, we observed an imbalance between scaffold degradation kinetics, which is probably due to macrophage-induced enhanced oxidative degradation, and tissue formation by the host. Henceforth, we intend to further optimize and strengthen the PCL2000-U4U, either by chemically adjusting its degradation kinetics, or by incorporating bioactive factors to enhance tissue formation. Future studies will also include longer follow-up times to evaluate the development in the long-term.

## Supplementary Materials

The following are available online at http://www.mdpi.com/2306-5354/5/3/61/s1, Table S1: Genes analyzed by quantitative real-time polymerase chain reaction.

## References

[1] Bouten C.V.C., Dankers P.Y.W., Driessen-Mol A., Pedron S., Brizard A.M.A., Baaijens F.P.T. (2011). Substrates for cardiovascular tissue engineering. Adv. Drug Del. Rev..

[2] Rocco K.A., Maxfield M.W., Best C.A., Dean E.W., Breuer C.K. (2014). In vivo applications of electrospun tissue-engineered vascular grafts: A review. Tissue Eng. Part B Rev..

[3] Enoch S., Leaper D.J. (2008). Basic science of wound healing. Surgery.

[4] Roh J.D., Sawh-Martinez R., Brennan M.P., Jay S.M., Devine L., Rao D.A., Yi T., Mirensky T.L., Nalbandian A., Udelsman B. (2010). Tissue-engineered vascular grafts transform into mature blood vessels via an inflammation-mediated process of vascular remodeling. Proc. Natl. Acad. Sci. USA.

[5] Hibino N., Yi T., Duncan D.R., Rathore A., Dean E., Naito Y., Dardik A., Kyriakides T., Madri J., Pober J.S. (2011). A critical role for macrophages in neovessel formation and the development of stenosis in tissue-engineered vascular grafts. FASEB J..

[6] Talacua H., Smits A.I.P.M., Muylaert D.E.P., van Rijswijk J.W., Vink A., Verhaar M.C., Driessen-Mol A., van Herwerden L.A., Bouten C.V.C., Kluin J. (2015). In situ tissue engineering of functional small-diameter blood vessels by host circulating cells only. Tissue Eng. Part A.

[7] de Valence S., Tille J.-C., Mugnai D., Mrowczynski W., Gurny R., Möller M., Walpoth B.H. (2012). Long term performance of polycaprolactone vascular grafts in a rat abdominal aorta replacement model. Biomaterials.

[8] Wu W., Allen R.A., Wang Y. (2012). Fast-degrading elastomer enables rapid remodeling of a cell-free synthetic graft into a neo-artery. Nat. Med..

[9] Pektok E., Nottelet B., Tille J.-C., Gurny R., Kalangos A., Moeller M., Walpoth B.H. (2008). Degradation and healing characteristics of small-diameter poly(epsilon-caprolactone) vascular grafts in the rat systemic arterial circulation. Circulation.

[10] Tara S., Kurobe H., Maxfield M.W., Rocco K.A., Yi T., Naito Y., Breuer C.K., Shinoka T. (2015). Evaluation of remodeling process in small-diameter cell-free tissue-engineered arterial graft. J. Vasc. Surg..

[11] Sugiura T., Tara S., Nakayama H., Kurobe H., Yi T., Lee Y.U., Lee A.Y., Breuer C.K., Shinoka T. (2016). Novel bioresorbable vascular graft with sponge-type scaffold as a small-diameter arterial graft. Ann. Thorac. Surg..

[12] Bergmeister H., Seyidova N., Schreiber C., Strobl M., Grasl C., Walter I., Messner B., Baudis S., Fröhlich S., Marchetti-Deschmann M. (2015). Biodegradable, thermoplastic polyurethane grafts for small diameter vascular replacements. Acta Biomater..

[13] Brunsveld L., Folmer B.J.B., Meijer E.W., Sijbesma R.P. (2001). Supramolecular polymers. Chem. Rev..

[14] Dankers P.Y.W., Harmsen M.C., Brouwer L.A., van Luyn M.J.A., Meijer E.W. (2005). A modular and supramolecular approach to bioactive scaffolds for tissue engineering. Nat. Mater..

[15] van Almen G.C., Talacua H., Ippel B.D., Mollet B.B., Ramaekers M., Simonet M., Smits A.I.P.M., Bouten C.V.C., Kluin J., Dankers P.Y.W. (2016). Development of non-cell adhesive vascular grafts using supramolecular building blocks. Macromol. Biosci..

[16] Muylaert D.E.P., van Almen G.C., Talacua H., Fledderus J.O., Kluin J., Hendrikse S.I.S., van Dongen J.L.J., Sijbesma E., Bosman A.W., Mes T. (2016). Early in-situ cellularization of a supramolecular vascular graft is modified by synthetic stromal cell-derived factor-1α derived peptides. Biomaterials.

[17] Kluin J., Talacua H., Smits A.I.P.M., Emmert M.Y., Brugmans M.C.P., Fioretta E.S., Dijkman P.E., Söntjens S.H.M., Duijvelshoff R., Dekker S. (2017). In situ heart valve tissue engineering using a bioresorbable elastomeric implant—From material design to 12 months follow-up in sheep. Biomaterials.

[18] Wisse E., Spiering A.J.H., van Leeuwen E.N.M., Renken R.A.E., Dankers P.Y.W., Brouwer L.A., van Luyn M.J.A., Harmsen M.C., Sommerdijk N.A.J.M., Meijer E.W. (2006). Molecular recognition in poly(ε-caprolactone)-based thermoplastic elastomers. Biomacromolecules.

[19] Zilla P., Bezuidenhout D., Human P. (2007). Prosthetic vascular grafts: Wrong models, wrong questions and no healing. Biomaterials.

[20] Pennel T., Zilla P., Bezuidenhout D. (2013). Differentiating transmural from transanastomotic prosthetic graft endothelialization through an isolation loop-graft model. J. Vasc. Surg..

[21] Mantovani A., Biswas S.K., Galdiero M.R., Sica A., Locati M. (2013). Macrophage plasticity and polarization in tissue repair and remodeling. J. Pathol..

[22] Garg K., Pullen N.A., Oskeritzian C.A., Ryan J.J., Bowlin G.L. (2013). Macrophage functional polarization (M1/M2) in response to varying fiber and pore dimensions of electrospun scaffolds. Biomaterials.

[23] Ballotta V., Driessen-Mol A., Bouten C.V.C., Baaijens F.P.T. (2014). Strain-dependent modulation of macrophage polarization within scaffolds. Biomaterials.

[24] Brown B.N., Ratner B.D., Goodman S.B., Amar S., Badylak S.F. (2012). Macrophage polarization: An opportunity for improved outcomes in biomaterials and regenerative medicine. Biomaterials.

[25] MacMicking J., Xie Q.W., Nathan C. (1997). Nitric oxide and macrophage function. Annu. Rev. Immunol..

[26] Brugmans M.C.P., Söntjens S.H.M., Cox M.A.J., Nandakumar A., Bosman A.W., Mes T., Janssen H.M., Bouten C.V.C., Baaijens F.P.T., Driessen-Mol A. (2015). Hydrolytic and oxidative degradation of electrospun supramolecular biomaterials: In vitro degradation pathways. Acta Biomater..

[27] Tille J.-C., de Valence S., Mandracchia D., Nottelet B., Innocente F., Gurny R., Möller M., Walpoth B.H. (2016). Histologic assessment of drug-eluting grafts related to implantation site. J. Dev. Biol..

[28] Sordi V., Malosio M.L., Marchesi F., Mercalli A., Melzi R., Giordano T., Belmonte N., Ferrari G., Leone B.E., Bertuzzi F. (2005). Bone marrow mesenchymal stem cells express a restricted set of functionally active chemokine receptors capable of promoting migration to pancreatic islets. Blood.

[29] Thevenot P.T., Nair A.M., Shen J., Lotfi P., Ko C.Y., Tang L. (2010). The effect of SDF-1alpha into PLGA scaffolds on stem cell recruitment and the inflammatory response. Biomaterials.

[30] Goumans M.-J., Liu Z., ten Dijke P. (2009). TGF-beta signaling in vascular biology and dysfunction. Cell Res..

[31] Wynn T.A., Ramalingam T.R. (2012). Mechanisms of fibrosis: Therapeutic translation for fibrotic disease. Nat. Med..

[32] Sugiura T., Tara S., Nakayama H., Yi T., Lee Y.-U., Shoji T., Breuer C.K., Shinoka T. (2017). Fast-degrading bioresorbable arterial vascular graft with high cellular infiltration inhibits calcification of the graft. J. Vasc. Surg..

[33] Wagenseil J.E., Mecham R.P. (2009). Vascular extracellular matrix and arterial mechanics. Physiol. Rev..

[34] Naito Y., Williams-Fritze M., Duncan D.R., Church S.N., Hibino N., Madri J.A., Humphrey J.D., Shinoka T., Breuer C.K. (2012). Characterization of the natural history of extracellular matrix production in tissue-engineered vascular grafts during neovessel formation. Cells Tissues Organs.

[35] Naito Y., Lee Y.-U., Yi T., Church S.N., Solomon D., Humphrey J.D., Shinoka T., Breuer C.K. (2014). Beyond burst pressure: Initial evaluation of the natural history of the biaxial mechanical properties of tissue-engineered vascular grafts in the venous circulation using a murine model. Tissue Eng. Part. A.

[36] Huang A.H., Balestrini J.L., Udelsman B.V., Zhou K.C., Zhao L., Ferruzzi J., Starcher B.C., Leven M.J., Humphrey J.D., Niklason L.E. (2016). Biaxial stretch improves elastic fiber maturation, collagen arrangement, and mechanical properties in engineered arteries. Tissue Eng. Part C Methods.

[37] Udelsman B.V., Khosravi R., Miller K.S., Dean E.W., Bersi M.R., Rocco K., Yi T., Humphrey J.D., Breuer C.K. (2014). Characterization of evolving biomechanical properties of tissue engineered vascular grafts in the arterial circulation. J. Biomech..

[38] Khosravi R., Miller K.S., Best C.A., Shih Y.C., Lee Y.U., Yi T., Shinoka T., Breuer C.K., Humphrey J.D. (2015). Biomechanical diversity despite mechanobiological stability in tissue engineered vascular grafts two years post-implantation. Tissue Eng. Part. A.

