# Supplementary material for: Host Response and Neo-Tissue Development during Resorption of a Fast Degrading Supramolecular Electrospun Arterial Scaffold
Source: Bioengineering (Basel). 2018 Aug 6;5(3):61. doi: 10.3390/bioengineering5030061 (PMC6164451; doi:10.3390/bioengineering5030061)
Supplement: Supplementary file 1 [file bioengineering-05-00061-s001.pdf]

Table S1. Genes analyzed by quantitative real-time polymerase chain reaction.

**Primer**	**Symbol**	**Accession number**	**Primer sequence (5'-3')**
Topoisomerase (DNA) I	TOP1	n/a	Primerdesign
β-2 microglobulin	B2M	n/a	Primerdesign
ATP synthase, H+ transporting, mitochondrial F1 complex, beta polypeptide	ATP5B	n/a	Primerdesign
Monocyte Chemotactic Protein-1	MCP-1	NM_031530	FW: CAGGTCTCTGTCACGCTTCT<br>RV: TGCTGCTGGTGATTCTCTTGT
Transforming Growth Factor-β	TGF-β	NM_021578.2	FW: TCAGACATTCGGGAAGCAGT<br>RV: GACAGCCACTCAGGCGTATC
Stromal cell-Derived Factor-1α	SDF-1α	NM_022177	FW: GCTCTGCATCAGTGACGGT<br>RV: CTGGCGACATGGCTCTCAA
Inducible Nitric Oxide Synthase	iNOS	NM_012611.3	FW: CTTGGTGAGGGGACTGGACTTTTAG<br>RV: GCCATGTCTGTGACTTTGTGCT
Arginase 1	Arg 1	NM_017134.3	FW: TGGTAGCAGAGACCCAGAAGA<br>RV: GAGCATCCACCCAAATGACG
Mannose receptor	MR	NM_001106123	FW: AAGCCTGTAGGAAGGAGGGT<br>RV: TCCCATCGCTCCACTCAAAG
Interleukin 1β	IL-1β	NM_031512.2	FW: GCAGGCTTCGAGATGAACAAC<br>RV: TTGTCGTTGCTTGTCTCTCCT
Interleukin 4	IL-4	NM_201270	FW: CACTTTGAACCAGGTCACAGA<br>RV: CTCGTTCTCCGTGGTGTTC
Interleukin 6	IL-6	NM_012589.2	FW: TCTGGTCTTCTGGAGTTCCGT

			RV: GGAAGTTGGGGTAGGAAGGAC
Interleukin 13	IL-13	NM_053828.1	FW: GAGCAACATCACACAAGACCAG<br>RV: TGGAGATGTTGGTCAGGGATT
Interferon-γ	IFN-γ	NM_138880	FW: ATGGATGCTATGGAAGGAAAGAG<br>RV: AGAGTCTGAGGTAGAAAGAGATAATC
Collagen I	Col I	NM_053304.1	FW: TGACGCATGGCCAAGAAGAC<br>RV: ATCAGGTTTCCACGTCTCACC
Collagen III	Col III	NM_032085.1	FW: TTCCTGGGAGAAATGGCGAC<br>RV: CCAGGATAGCCACCCATTCC
Collagen IV	Col IV	NM_001135009.1	FW: GGCAGAGGAACGTGCAATTAC<br>RV: GCGTGGGCTTCTTGAACATC
Elastin	Ela	NM_012722.1	FW: AGTTCCTGGTGTCGGTCTTC<br>RV: ACCTTGGCCTTGACTCCTGT
